# Exogenous Glutathione Alleviates Cadmium Toxicity in Wheat by Influencing the Absorption and Translocation of Cadmium

**DOI:** 10.1007/s00128-021-03283-8

**Published:** 2021-06-10

**Authors:** Ge-Zi Li, Shi-Juan Chen, Na-Ying Li, Ying-Ying Wang, Guo-Zhang Kang

**Affiliations:** 1 National Engineering Research Center for Wheat, Henan Agricultural University, #15 Longzihu College District, Zhengzhou, 450046 Henan Province People’s Republic of China; 2 National Key Laboratory of Wheat and Maize Crop Science, Henan Agricultural University, #15 Longzihu College District, Zhengzhou, 450046 Henan Province People’s Republic of China

**Keywords:** *Triticum aestivum* L., Cd, GSH, GSH synthesize genes, Cd transporter genes

## Abstract

Cadmium (Cd), a toxic heavy metal, is harmful to plants and human health. Glutathione (GSH) could alleviate Cd toxicity of plant species, whereas its mechanism responsible for wheat remains poorly understood. Here, we found that exogenous GSH application significantly increased the fresh and dry weight, root elongation, chlorophyll contents, while decreased the contents of malondialdehyde (MDA) and GSH, and translocation factor of Cd compared with Cd treatment. Moreover, GSH application significantly increased activities of antioxidant enzymes and expression of related genes, which involved in GSH synthesis, especially in roots. In addition, we found that GSH application suppressed Cd-induced expression of metal transporter genes *TaNramp1*, *TaNramp5*, *TaHMA2*, *TaHMA3*, *TaLCT1* and *TaIRT2* in roots. Taken together, our results suggested that GSH could alleviate Cd toxicity in wheat by increasing GSH synthesis gene expression or suppressing Cd transporter genes expression, and further affecting Cd uptake and translocation in wheat plants.

**Supplementary Information:**

The online version contains supplementary material available at 10.1007/s00128-021-03283-8.

Cadmium (Cd), a toxic heavy metal, is particularly dangerous and widely spread in the soil, and its accumulation would inhibit plant growth and development by disrupting the enzyme activities, hormone balance, protein synthesis, and key metabolic reactions (Rizwan et al. 2016a). It has reported that Cd accumulation in plants decreases crop production and quality, meanwhile, it also threatens human health (such as leading to cancer, cardiovascular, and osteoporosis disease) by food chain (Aziz et al. 2015). Therefore, it is imperative to block the Cd uptake and translocation from polluted soil to the edible parts of crops.

Plants have developed ranges of mechanisms to resist Cd toxicity, such as reducing Cd uptake and translocation, and detoxifying/sequestering Cd in the subcellular organs (Liu et al. 2020; Jiang et al. 2020). Glutathione (GSH), a kind of non-enzymatic antioxidant from ascorbic acid-glutathione (ASA-GSH) system, is a low molecular weight thiol compound in plants, playing an important role in the different biological processes (Kuźniak et al. 2013), such as participating in the enzymatic reaction to reduce accumulation of reactive oxygen species and maintaining redox status homeostasis (Szalai et al. 2009). Furthermore, it also involves in phytochelatin synthesis (PCs), which could form complexes with the excessive heavy metals in the cytosol and then transported into vacuoles to alleviate toxicity of plants (Seth et al. 2012). Moreover, GSH participates in signal transduction of plant cells by controlling the gene expression in the processes of various abiotic stress, including heavy metals detoxification (Kim et al. 2017). However, the mechanism of GSH alleviated Cd tolerance in wheat still remains unclear.

Cd uptake, translocation, and detoxification in plants were carried out by a series of signal transduction pathways. For instance, several mediated Cd uptake and translocation genes, including Natural Resistance-Associated Macrophage Protein (NRAMP) family, Iron-Regulated Transporter 1 (IRT1) homologs, Low affinity Cation Transporter (LCT), and Heavy Metal ATPase (HMA), which were identified and characterized for the function mediated-Cd uptake or translocation in plants (Takahashi et al. 2012). On the other hand, plants also response to Cd stress by inducing the expression of defense genes, which contains antioxidants comprising ROS-removing enzyme gene ascorbate peroxidase (APX), glutathione reductase (GR), and non-enzymatic system genes GSH, glutathione S-transferase (GST), dehydroascorbate reductase (DHAR), and monodehydroascorbate reductase (MDHAR) in ASA-GSH system (Qin et al. 2018). However, the precise regulatory pathways influencing the above transporter or defense genes involved in Cd uptake and allocation are poorly clear.

Wheat, one of the globally important crops, has high and stable yield for humans and livestock, and thus, it is of great significance to the food security of China (Chen et al. 2019). Compared with other cereals, in Cd contaminated soils, wheat can accumulate more Cd, mainly through the roots, which is transferred to aerial parts of the plant, and finally accumulated in the wheat grains, and the products of wheat are the major source of Cd intake by humans (Li et al. 2020). Therefore, an important demand is that Cd minimization in wheat especially in Cd-contaminated soils. To date, however, there were no studies reported that GSH mediated-Cd tolerance in wheat. To further study the effects of exogenous GSH on Cd tolerance in wheat, the characteristics of plant growth, the enzyme activities of GSH metabolism, the transcription levels of several genes involved in Cd uptake, detoxification and accumulation, and the uptake or accumulation of Cd elements, have been analyzed in this study. All results suggested that external GSH could alleviate Cd toxicity in wheat, and these results from our study will improve the understanding of Cd tolerance mechanisms in wheat and help develop strategies for alleviating Cd toxicity in wheat cultivation.

## Materials and Methods

Wheat seeds (*Triticum aestivum* L. cv. Bainong 207) were surface-sterilized and germinated as previously described in our published literature (Li et al. 2013). Firstly, wheat seedlings grew in 1/2-strength Hoagland’s solution (pH 6.5) for two weeks (Li et al. 2017a, b). These two-week-old wheat seedlings were transferred to 0.5 mM CaCl_2_ solution (pH 4.5), which could reduced the competition of ions in Hoagland's solution and provided an acidic environment for increasing the activity of Cd^2+^ (Corrêa et al., 2011; Liu et al., 2018). Then, they were treated for 10 d, which was selected according to the effect of different durations during Cd stress of wheat seedlings (Figure S1), with CK (0.5 mM CaCl_2_), Cd (0.5 mM CaCl_2_ + 50 μΜ CdCl_2_), Cd + GSH (0.5 mM CaCl_2_ + 50 μΜ CdCl_2_ + 20 μΜ GSH), and GSH (0.5 mM CaCl_2_ + 20 μM GSH). And the concentrations of Cd and GSH were performed as previously studies (Rizwan et al. 2016b; Li et al. 2017a, b; Qin et al. 2018). All these solutions were renewed every 48 h.

The plant growth parameters, such as root length, fresh weight, dry weight, were measured after 10 d of treatment. The relative root elongation (RRE) and chlorophyll contents were calculated according to the method of Zheng et al. (2005) and Li et al. (2013). For root scanning, the whole roots were collected and scanned by flatbed scanner (Epson expression 1680 1.0 scanner, Japan). And the others samples were stored at – 80°C.

Plant shoots and roots were dried and digested as previous study (Aprile et al. 2018), then, the shoot/root translocation factors of Cd were calculated according to the method of Zhu et al. (2020). Moreover, Cd contents in plant subcellular fractions, containing cell walls, organelles and cytosol, were also measured according to the method of Wu et al. (2005).

The malondialdehyde (MDA) and reduced glutathione (GSH) content assays according to our previous study (Li et al. 2013). The activity of glutathione reductase coefficient (GR), glutathione S-transferase (GST) and glutathione peroxidase (APX) by using Assay Kits (Nanjing Jiancheng Institute of Biological Engineering, China).

Total RNA of wheat shoots and roots were extracted by using TransZol RNA Isolation Reagent (Tiangen, China) and determined the concentration. Then, 1 μg of total RNA was prepared for reverse transcription by using ReverTra Ace qPCR RT Kit (Toyobo, Japan). qPCR was performed using AceQ™ Universal SYBR® qPCR Master Mix (Vazyme, China) as following reaction conditions: one cycle at 95°C for 10 min, 40 cycles of amplification at 95°C for 10 s, 58°C for 15 s, and finally 72°C for 20 s in a QuantStudio 3 (Thermo, USA) system. The relative expression levels were calculated according to the method of 2^−ΔΔCT^ (Schmittgen and Livak 2008). Wheat Glyceraldehyde 3-phosphate dehydrogenase (*GAPDH*) gene was used as an endogenous control gene in this study. All used primers were listed in Table S1.

Statistically analyses were performed by using the SPSS software (version 25.0), and the statistical assays were tested with LSD-test to evaluate significant differences between the treatments using a significance threshold of *p* < 0.05. All the data in this study was calculated at least three biological replicates.

## Results and Discussion

To determine the effect of GSH on Cd toxicity, wheat seedlings grew in different culture medium for 10 days. Compared with non-treated control (CK), the growth of wheat plants was inhibited, the color of leaves were displayed visible chlorosis, and the length of roots were inhibited in Cd treatment, while exogenous GSH significantly alleviated the above Cd toxicity effect (Figure S2). After application with exogenous GSH, the chlorophyll a and b contents restored to 61.6% and 65.9% of CK, respectively; and it was greater 2.35- and 2.42-folds than only treated with Cd (Table 1); the relative root elongation was inhibited 84.62% of CK, and it was lower 2.0-folds than that of only Cd treatment (Table 1). Similarly, the fresh and dry biomass of plants, which were treated with Cd + GSH treatment, decreased by 37.55% and 25.85% of CK, and they were 1.41- and 1.17-folds higher than that of Cd treatments (Table 1).

**Table 1:** Chlorophyll contents and biomass of wheat seedlings with or without Cd or GSH

Treatments	Chlorophyll a content (mg·g^−1^)	Chlorophyll b content (mg·g^−1^)	RRE (%)	Fresh weight (g·plant^−1^)	Dry weight (g·plant^−1^)
CK	1.18 ± 0.002^a^	0.44 ± 0.002^b^	100.0 ± 0.455^a^	0.77 ± 0.087^a^	0.079 ± 0.01^a^
GSH	0.82 ± 0.001^b^	0.68 ± 0.002^a^	74.36 ± 0.586^b^	0.64 ± 0.073^b^	0.073 ± 0.01^b^
Cd	0.31 ± 0.002^d^	0.12 ± 0.001^d^	7.700 ± 0.208^d^	0.37 ± 0.041^d^	0.050 ± 0.01^d^
Cd + GSH	0.73 ± 0.001^c^	0.29 ± 0.002^c^	15.38 ± 0.830^c^	0.52 ± 0.030^c^	0.058 ± 0.01^c^

RRE, Relative root elongation; Data present means ± SE (n = 3). The different letters represent significant difference at *p* < 0.05

**Fig. 1:**
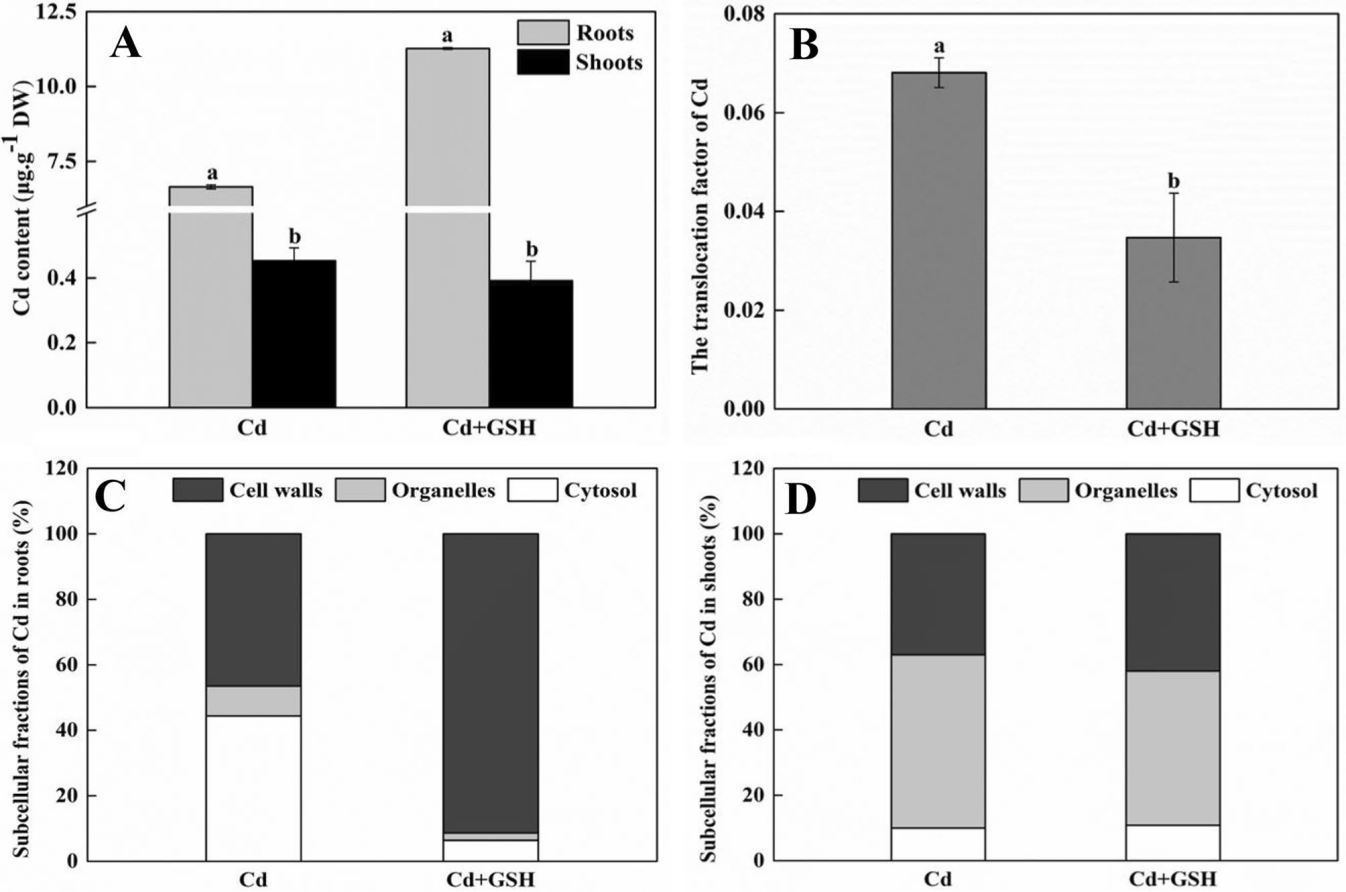
Cd content in wheat seedlings after Cd and GSH treatment for 10 days. **a** The content of Cd in roots and shoots of wheat seedlings; **b** The translocation factor of Cd from roots to shoots; **c** and **d** The subcellular distribution of Cd in roots and shoots of wheat seedlings, respectively. Data present means ± SE (n = 3). The different letters represent significant difference at *p* < 0.05

Supplement of GSH could modified metal toxicity by altering the rates of metal uptake or chelating metal ions in direct and indirect ways in different plants, including barley, rice, and maize (Chen et al. 2011; Cao et al. 2015; Li et al. 2017a, b). Similarly, in our study, application with exogenous GSH increased by 69.55% of Cd content in roots and decreased by 15.78% of Cd content in shoots, respectively (Fig. 1a). However, the translocation factor of Cd was decreased 0.49-fold in present with exogenous GSH compared with only Cd treatments (Fig. 1b). Further, the Cd contents in the fractions of cell walls (Fig. 1c), which are used for Cd sequestered and have a low toxicity, were significantly increased 260.5% in roots by GSH. While, in roots or shoots, the Cd levels were both decreased 55.3% or 34.1% and 19.8% or 73.4% by GSH in the organelles and cytosol (Fig. 1c and d), where the liable Cd is most toxic. These results indicated that exogenous GSH decreased root-to-shoot Cd translocation by sequestering more Cd in cell walls and regulated the subcellular compartmentalization of Cd in wheat roots.

To verify the role of exogenous GSH ameliorated oxidative stress under Cd stress, some indexes of oxidative stress, which contained the contents of MDA or GSH, and anti-oxidant enzymes (GST, APX, and GR), were determined in wheat plants. The results showed that exogenous GSH supplement significantly decreased 1.85- or 1.65-folds of MDA or reduced GSH contents compared with Cd treatment in roots, while they did not significantly changed in shoots (Table 2). Moreover, exogenous GSH supplement were significantly increased 32.4% and 12.9% of GST and GR activity compared with Cd treatment in roots (Table 3), respectively. And it significantly decreased the APX activity by 7.6% and 13.4% in roots and shoots, and GR activity by 21.8% in shoots compared with Cd treatment, respectively (Table 3). Compared with CK, GSH supplement had no effect on GST activity in shoots (Table 3). Similarly, application of exogenous GSH reduced contents of reactive oxidative species and MDA in maize, rice and tomato under Cd stress (Hasan et al. 2016; Li et al. 2017a, b; Zhou et al. 2017).

**Table 2:** Contents of MDA and reduced GSH in wheat seedlings

Treatments	MDA content (μmol·g^−1^ FW)		Reduced GSH content (μmol·g^−1^ FW)	
	Roots	Shoots	Roots	Shoots
CK	0.91 ± 0.04^c^	9.66 ± 0.17^d^	0.79 ± 0.01^d^	1.89 ± 0.01^a^
GSH	0.93 ± 0.07^c^	10.89 ± 0.30^c^	0.82 ± 0.05^c^	1.68 ± 0.03^c^
Cd	2.16 ± 0.17^a^	22.88 ± 0.80^a^	3.02 ± 0.06^a^	1.73 ± 0.01^b^
Cd + GSH	1.17 ± 0.01^b^	19.21 ± 1.19^b^	1.83 ± 0.05^b^	1.74 ± 0.04^b^

**Table 3:** Activity of antioxidant enzymes in wheat seedlings

Treatments	GST activity (μmol·mg^−1^ protein min^−1^)		APX activity (μmol·mg^−1^ protein min^−1^)		GR activity (μmol·mg^−1^ protein min^−1^)	
	Roots	Shoots	Roots	Shoots	Roots	Shoots
CK	133.78 ± 9.6^c^	45.15 ± 1.2^a^	122.66 ± 4.4^c^	41.57 ± 0.9^c^	0.54 ± 0.0^d^	1.17 ± 0.1^a^
GSH	149.11 ± 2.9^c^	38.51 ± 2.0^b^	123.76 ± 2.3^c^	45.55 ± 0.7^b^	0.90 ± 0.1^c^	1.02 ± 0.0^b^
Cd	170.40 ± 5.5^b^	46.78 ± 1.9^a^	211.06 ± 3.7^a^	53.38 ± 1.1^a^	1.15 ± 0.0^b^	1.10 ± 0.0^a^
Cd + GSH	225.53 ± 5.6^a^	49.29 ± 2.3^a^	195.02 ± 1.7^b^	46.24 ± 0.6^b^	1.30 ± 0.0^a^	0.86 ± 0.0^c^

**Fig. 2:**
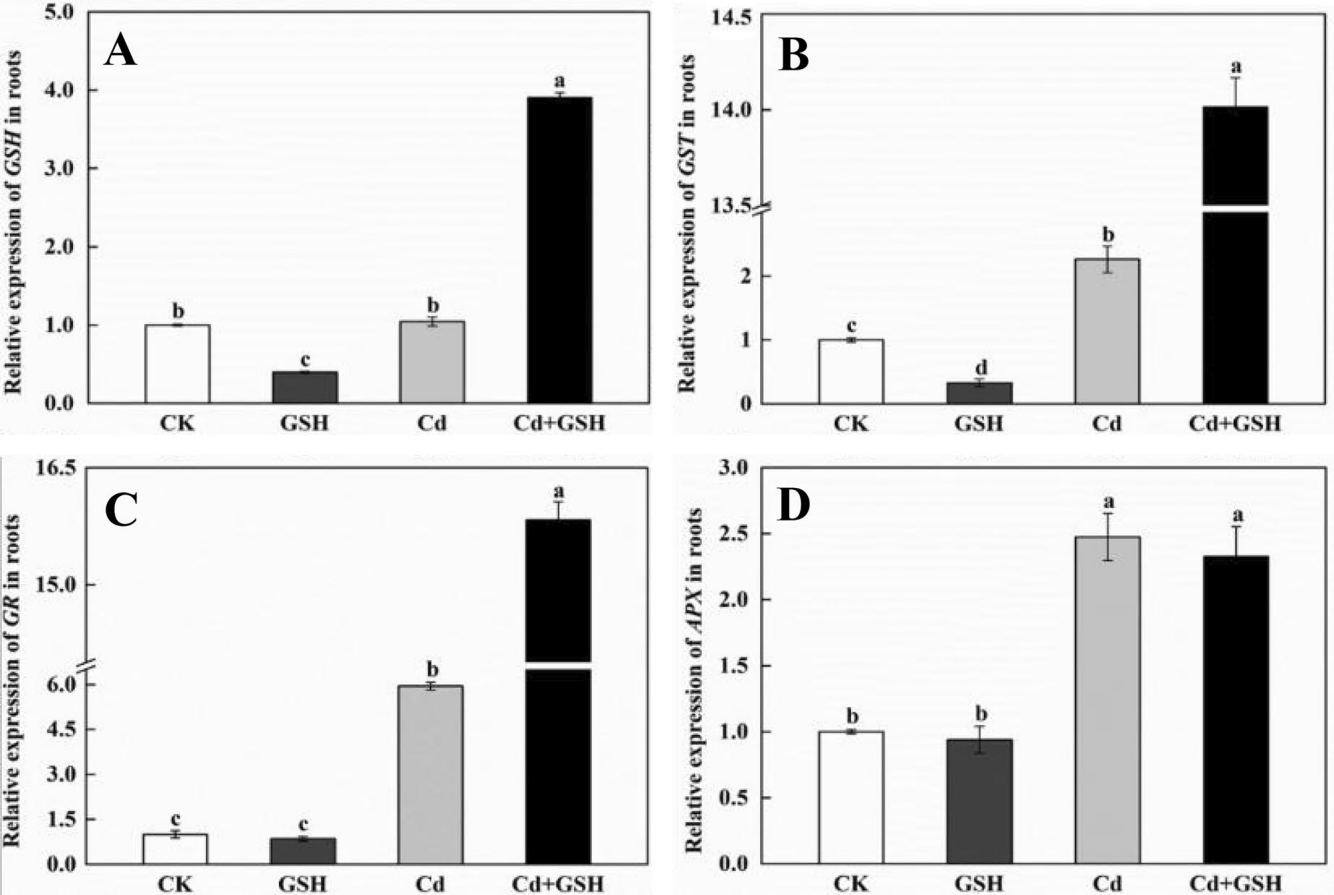
Relative expression levels of genes in ASA-GSH cycles after Cd and GSH treatment for 10 days. The expression levels of *GSH* (**a**)*, GST* (**b**)*, GR* (**c**)*,*and *APX* (**d**) in roots of wheat seedlings; Data present means ± SE (n = 3). The different letters represent significant difference at *p* < 0.05

Transcription of defense gene expression provided more precise estimation than their enzyme activity in response to abiotic stresses (Chen et al. 2011; Kang et al. 2013). Therefore, the expression levels of four GSH synthesis genes were determined to investigate the molecular mechanism of GSH regulating Cd tolerance in wheat plants. Compared with CK, the expression levels of *GSH*, *GST*, *GR* and *APX* were significantly induced 1.04, 2.26, 5.94, 2.48-folds in roots (Fig. 2) or 1.83, 3.68, 2.20, 1.25-folds in shoots (Figure S3) under Cd treatment, respectively. Exogenous GSH application were further enhanced the expression levels of *GSH* (3.74-fold), *GST* (6.19-fold), *GR* (2.66-fold) in roots (Fig. 2a–c) or *APX* (1.79-fold) in shoots (Figure S3D), while significantly decreased the expression levels of *GSH* (46.3%), *GST* (19.8%), *GR* (79.9%) in shoots (Figure S3B, 3D and 3F), compared with Cd treatment. This suggested that exogenous GSH could differentially regulated the expression profiles of these genes which might induced the changes of GSH content under Cd stress.

**Fig. 3:**
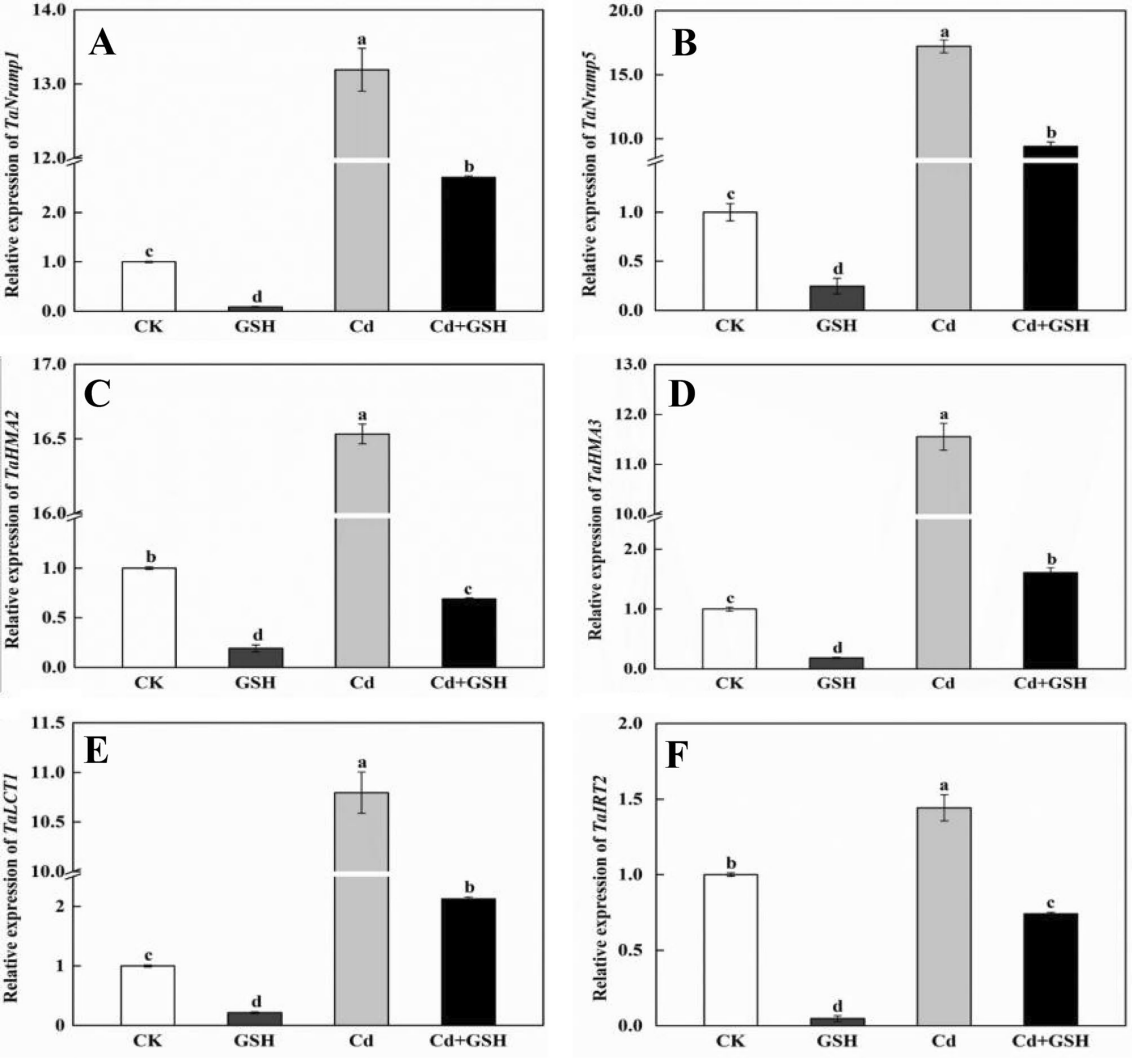
Relative expression levels of Cd transporter genes in roots of wheat seedlings. The expression levels of *TaNramp1* (**a**), *TaNramp5* (**b**), *TaHMA2* (**c**), *TaHAM3* (**d**), *TaLCT1* (**e**), and *TaIRT2* (**f**) genes. Data present means ± SE (n = 3). The different letters represent significant difference at *p* < 0.05

Regulation and manipulation of metal transporters is an important mechanism of alleviating heavy metal toxicity in plants (Ueno et al. 2010; Liu et al. 2013; Adrees et al. 2015). Such as the expression levels of *OsNramp1*, *OsNramp5*, *OsHMA2* and *OsHMA3*, which were increased in rice roots exposure to Cd stress (Chen et al. 2019). And Yang et al. (2019) found Lanthanum reduced Cd accumulation in wheat by down-regulation the expression levels of *TaNramp5* and *TaHMA2*. Consistent with these, the expression levels of these Cd transporters were elevated in wheat plants to further understand the molecular mechanism of how GSH regulates the absorption, translocation, and allocation of Cd in wheat plants. The results showed that the expression levels of *TaNramp1*, *TaNramp5*, *TaHMA2*, *TaHMA3*, *TaLCT1*, and *TaIRT2* were greatly induced 13.2-, 17.2-, 16.5-, 11.6-, 10.8- and 1.4-folds by Cd stress in roots compared with CK, respectively (Fig. 3). However, their transcriptions were significantly decreased 2.0- to 30.0-folds by exogenous GSH in roots (Fig. 3). Similarly, in shoots, except to *TaIRT2* and *TaHMA3*, the expression levels of *TaNramp1*, *TaNramp5*, *TaHMA2* and *TaLCT1* were separately induced 1.6-, 2.8-, 4.9- and 1.3-folds by Cd stress. And the exogenous GSH application was significantly decreased transcription of these genes (3.1-folds of *TaNramp1*, 1.2-folds of *TaNramp5*, 3.3-folds of *TaHMA2*, and 4.0-folds of *TaLCT1*) (Figure S4). It is interestingly that the only supplement of exogenous GSH inhibited (75%-95%) the transcription of these transporter genes in roots (Fig. 3), while induced 1.19- to 5.92-folds in shoots (Figure S4). These results were consisted with previous study (Kim et al. 2014; Clemens and Ma 2016; Chen et al. 2019). These results demonstrated that exogenous GSH reversed the Cd enhanced expression of transporter genes responsible for Cd uptake and translocation to alleviate Cd toxicity in wheat plants, especially in roots.

In conclusion, adding GSH significantly alleviates the inhibition of growth, reduces lipid peroxidation, and effectively protects wheat seedlings from Cd stress damage. This enhanced tolerance could be relevant to the increased transcription of the GSH cycle-related genes and increased GSH content and biosynthesis, and decreased the transcription of Cd transporter genes. Our results contribute to elucidation of the effect of GSH on the plant response to Cd stress.

## Supplementary Information

Below is the link to the electronic supplementary material.

Supplementary file1 (PDF 451 kb)
