# Supplementary material for: Exogenous Glutathione Alleviates Cadmium Toxicity in Wheat by Influencing the Absorption and Translocation of Cadmium
Source: Bull Environ Contam Toxicol. 2021 Jun 10;107(2):320–6. doi: 10.1007/s00128-021-03283-8 (PMC8346402; doi:10.1007/s00128-021-03283-8)
Supplement: Supplementary file 1 — Supplementary file1 (PDF 451 kb) [file 128_2021_3283_MOESM1_ESM.pdf]

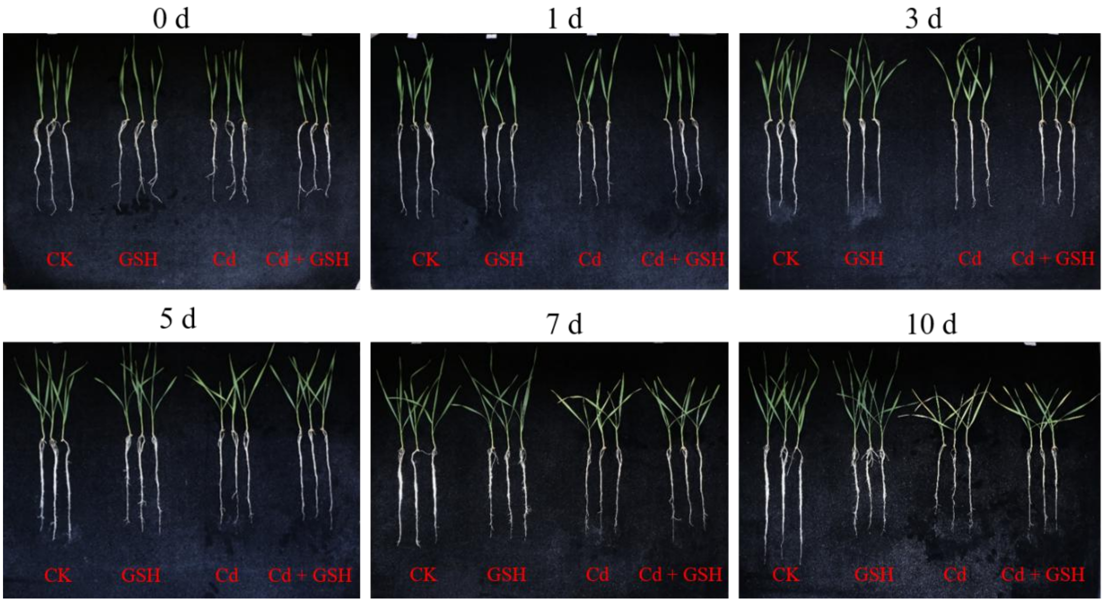

Figure S1. Effect of different durations of Cd stress on wheat seedlings. The phenotype of wheat seedlings were treated with CK (0.5mM CaCl2, pH 4.5), GSH (0.5mM $CaCl_2$ + 20 µM GSH), Cd (0.5mM $CaCl_2$ + 50 µM $CdCl_2$), or Cd + GSH for different durations (1 d, 2 d, 3 d, 5 d, 7 d, 10 d). The solution was renewed every 48 h.

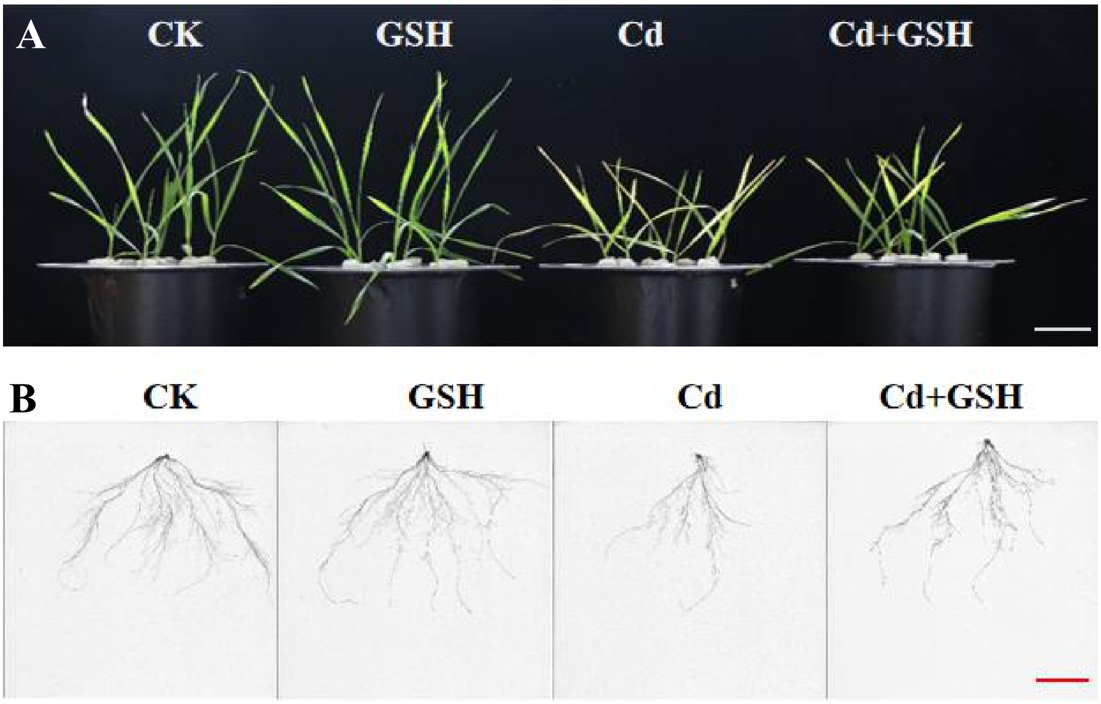

Figure S2. Effect of cadmium (Cd) and glutathione (GSH) supplementation on the growth of wheat seedlings. A, The phenotype of wheat seedlings were treated with CK (0.5mM $CaCl_2$, pH 4.5), GSH (0.5mM $CaCl_2$ + 20 µM GSH), Cd (0.5mM $CaCl_2$ + 50 µM $CdCl_2$), or Cd + GSH for 10 days. The solution was renewed every 48 h. Bar = 5.5 cm. B, The morphological structure of wheat under different treatment. Bar = 8.0 cm.

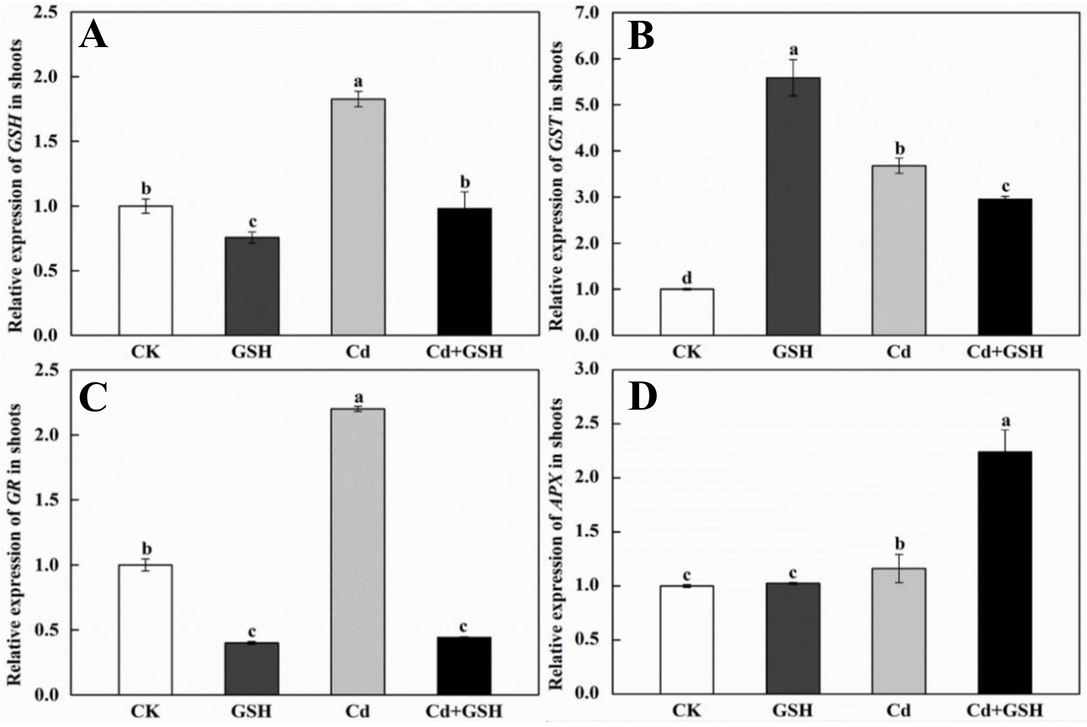

Figure S3. Relative expression levels of genes in ASA-GSH cycles after Cd and GSH treatment for 10days. The expression levels of *GSH* (A), *GST* (B), *GR* (C), and *APX* (D) in shoots of wheat seedlings; Data present means ± SE (n = 3). The different letters represent significant difference at $P < 0.05$.

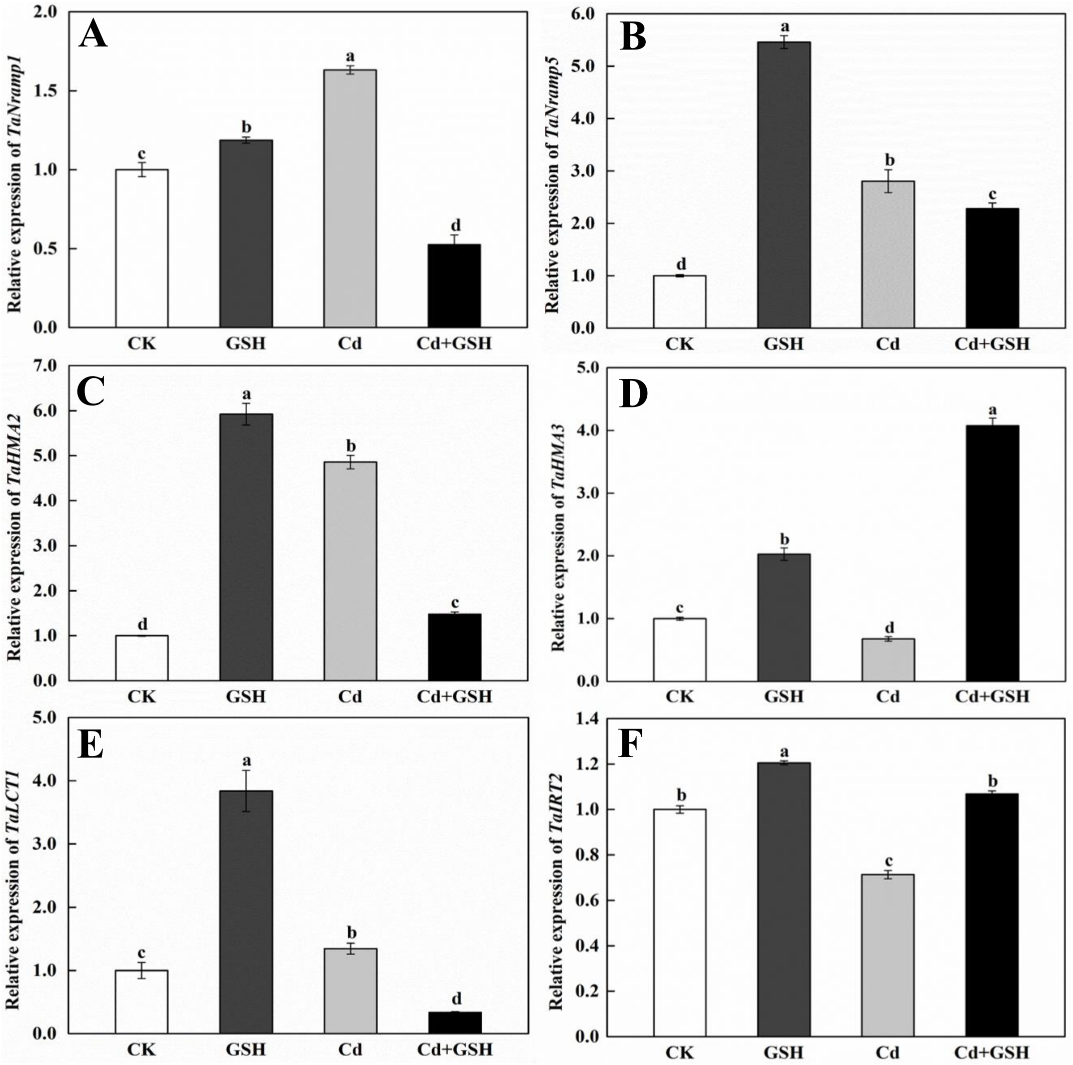

Figure S4. Relative expression levels of Cd transporter genes in shoots of wheat seedlings. A, The expression levels of *TaNramp1* gene; B, The expression levels of *TaNramp5* gene; C, The expression levels of *TaHMA2* gene; D, The expression levels of *TaHAM3* gene; E, The expression levels of *TaLCT1* gene; F, The expression levels of *TaIRT2* gene. Data present means ± SE (n = 3). The different letters represent significant difference at $P < 0.05$.

Table S1 Primers for qPCR

Gene name	NCBI No.	Primer sequence (5'-3')	Size (bp)
TaGR	AY364467	F: 5'-ATGAATACTCCCGTACATCAGT-3'<br>R: 5'-TTTGTTACATCACCCACAGC-3'	155
TaGST	AJ414697	F: 5'-TACGAGAGGTACGGCGAGTT-3'<br>R: 5'-CAGGCCGATGAAGTCGTACA-3'	135
TaGSH	AJ579382	F: 5'-ATCGCCAAGCTCCGTCAATG-3'<br>R: 5'-ACAAGTCAGGGTTTTCAATCG-3'	92
TaAPX	FJ890988	F: 5'-CTGAGTGGTGATAAAGAGGGA-3'<br>R: 5'-ACAAGAGGGCGGAAGACAG-3'	108
TaLCT1	AF015523	F: 5'-CATGAGGCCGACATCCTCTC-3'<br>R: 5'-TTCCTCTCAGTTGCACGACC-3'	156
TaHMA2	KF933095	F: 5'-GTCTCGCTCTTCGAGCACTT-3'<br>R: 5'-GCAACTGCGATGAGCATGAG-3'	149
TaHMA3	KF683297	F: 5'-GGGATGTCGTCGTTGATGAG-3'<br>R: 5'-ACCGTCCAAGTTGAGCGTG-3'	249
TaIRT2	AB126086	F: 5'-GTCGTCGTTCAGGTTCTGGA-3'<br>R: 5'-CCCATCCCCTCGAACATCTG-3'	140
TaNramp1	DQ431468	F: 5'-TATGGGGTACGGAAGCTGGA-3'<br>R: 5'-GAGCCTGGGGATGAATAGCC-3'	132
TaNramp5	AB698459	F: 5'-ACAGGAAAGCATCTTGCCGA-3'<br>R: 5'-ACAGGGATGCGGAACAAGAG-3'	155
TaGAPDH	KR029492	F: 5'-TTTTCACCGACAAGGACA-3'<br>R 5'-AAGAGGAGCAAGGCAGTT-3'	179

Notes, F, Forward primer; R, Reverse primer.
